# Rapid ion-exchange matrix removal for a decrease of detection limits in the analysis of salt-rich reservoir waters for fluorobenzoic acids by liquid chromatography coupled with tandem mass spectrometry

**DOI:** 10.1007/s00216-016-0060-5

**Published:** 2016-11-10

**Authors:** Paweł Kubica, Véronique Vacchina, Tomasz Wasilewski, Stéphanie Reynaud, Joanna Szpunar, Ryszard Lobinski

**Affiliations:** 1 Department of Analytical Chemistry, Faculty of Chemistry, Gdańsk University of Technology, Narutowicza 11/12, 80-233 Gdańsk, Poland; 2 UT2A, Hélioparc, 2, Avenue Pr. Angot, 64053 Pau, France; 3 CNRS-UPPA UMR 5254, Equipe de Physique et Chimie des Polymères (EPCP-IPREM), Hélioparc, 2, Avenue Pr. Angot, 64053 Pau, France; 4 CNRS-UPPA, UMR 5254, Laboratoire de Chimie Analytique Bio-inorganique et Environnement (LCABIE-IPREM), Hélioparc, 2, Avenue Pr. Angot, 64053 Pau, France; 5 Department of Analytical Chemistry, Warsaw University of Technology, ul. Noakowskiego 3, 00-664 Warsaw, Poland

**Keywords:** Fluorinated benzoic acids, LC MS/MS, Matrix removal, Ion exchange

## Abstract

A matrix removal procedure with ion-exchange resin prior to analysis for 18 fluorinated benzoic acids (FBAs) tracers in saline (>25% salt) reservoir water was optimized. The elimination of >98% of salt and the simultaneous matrix sample cleanup allowed the direct analysis using the supernatant by liquid chromatography coupled with tandem mass spectrometry (LC-MS/MS). This resulted in a gain in detection limits for most of the tracers in comparison with the reference method (direct analysis after minimum required dilution). The limits of detection (LODs) were in the range of 0.01–0.15 ng/ml and compared to other studies the developed method provided comparable limits of detection and advantage of simplified and shorter sample preparation. The presented method offers a considerable gain in simplicity and analysis time. Recoveries for all the tracers reached 80–100%, except for 2-FBA and 2,6-dFBA for which they were ca. 60%. The low recoveries were corrected by the use of five isotopically labeled internal standards. The method was validated by the analysis of spiked samples and by an independent comparison of the results with those obtained by solid-phase extraction LC-MS/MS method.

**Electronic supplementary material:**

The online version of this article (doi:10.1007/s00216-016-0060-5) contains supplementary material, which is available to authorized users.

## Introduction

Different fluorobenzoic acids are commonly used as non-radioactive passive tracers in petroleum exploration [1, 2]. Hence, there is a need for their sensitive analysis in oil reservoir waters, known for their highly dissolved organic matter and salt (NaCl, CaCl_2_) content often exceeding 25% [3]. Gas chromatography (GC) [4] or high-performance liquid chromatography (HPLC) [5–10] with MS/MS detection have been typically used to assure the separation of the tracer compounds from each other while assuring their specific detection.

HPLC-MS/MS offers detection limits down to 0.01 ng/ml for most of the fluorobenzoic acids in the selected reaction monitoring (SRM) mode using the ion transition corresponding to the loss of CO_2_ by the pseudomolecular ion employing the-state-of-the-art triple quadrupole, Q-TOF [7] or Q-Orbitrap [11] mass spectrometers. However, as the maximum tolerable salt content in the solution injected on the column used (Waters, Acquity UPLC BEH C18 column, 50 × 2.1 mm, 1.7 μm) has to be inferior to 1%, a dilution is required [10]. This represents for samples with 25% salinity a 25-fold increase in the detection limits making the method virtually useless for practical applications.

Sample preparation methods based on the solid-phase extraction (SPE) of fluorobezoic acids were therefore developed for the salt-removal and preconcentration of analytes. They allowed to obtain detection limits for salt-rich samples comparable with those obtained for standard solutions in water [4, 7, 8]. Moreover, methods presenting on-line SPE for determination of drugs or pesticides in different kind of sample are available as well [9, 10] However, the time necessary, relatively large volumes of organic solvents requiring evaporation made the procedures quite tedious for high-throughput analysis. Gas chromatography MS/MS suffers from similar drawbacks [11]. Although the detection limits are below 0.01 ng/mL, the required sample preparation procedures are time-consuming (24 h). Moreover, the derivatization step is incomplete and suffers from strongly compound-dependent yields which limits the practical use [11].

In contrast to all the reported protocols, until now [4, 7, 8], the objective of this method development was on the removal of the matrix rather than on the extraction of the analytes. The removal of salt matrix using mixed-bed ion-exchange resin was investigated. The proposed approach allows to remove matrix effects and thus resulting in a less noisy baseline and lower detection limits.

## Materials and methods

### Samples collection

Samples originated from reservoir waters (Congo) contained 250 g/L of total salt (primary Na and Ca with a minor contribution of Mg and K, while the major anion is Cl). The samples were collected in 5-L glass bottles. Sub-samples of 100 mL were transported in ambient temperature in glass flasks in containers preventing the exposure to light; the samples were acidified to approximately pH 2.40 with formic acid and stored prior to analysis at 4 °C in the dark. In these conditions, they were stable for at least 90 days.

### Standards

The FBA standards used in this study were as follows: 2-fluorobenzoic acid (2-FBA, purity 99%); 3-fluorobenzoic acid (3-FBA, purity 99%); 4-fluorobenzoic acid (4-FBA, purity 98%); 2,6-difluorobenzoic acid (2,6-dFBA, purity 98%); 2,5-difluorobenzoic acid (2,5-dFBA, purity 98%); 2,3-difluorobenzoic acid (2,3-dFBA, purity 98%); 2,4-difluorobenzoic acid (2,4-dFBA, purity 99%); 3,5-difluorobenzoic acid (3,5-dFBA, purity 97%); 3,4-difluorobenzoic acid (3,4-dFBA, purity 99%); 2,3,6-trifluorobenzoic acid (2,3,6-tFBA, purity 99%); 2,4,6-trifluorobenzoic acid (2,4,6-tFBA, 98%); 2,4,5-trifluorobenzoic acid (2,4,5-tFBA, purity 99.5%); 2,3,4-trifluorobenzoic acid (2,3,4-tFBA, purity 98%); 3,4,5-trifluorobenzoic acid (3,4,5-tFBA, purity 98%); 2-trifluoromethylbenzoic acid (2tFmBA, purity 98%); 3-trifluoromethylbenzoic acid (3-tFmBA, purity 99%); 4-trifluoromethylbenzoic acid (4-tFmBA, purity 98%); and 3,5-bis-trifluoromethylbenzoic acid (3,5-bisFmBA, purity 98%). They were purchased from Sigma-Aldrich (Saint-Quentin-Fallavier, France) (4FBA; 2,3dFBA; 3,5dFBA; 2,3,6-tFBA; 2,4,6-tFBA; 2,3,4-tFBA; 3,4,5-tFBA; 3-tFmBA; 4-tFmBA; and 3,5-bistFmBA) and Across Organics (supplied by Fisher Scientific SAS, Illkirch, France) (2-FBA; 3-FBA; 2,6-dFBA; 2,5-dFBA; 2,4-dFBA; 3,4-dFBA; 2,4,5-tFBA; and 2-tFmBA).

### Isotopically labeled standards

Deuterated sulfuric acid-d_2_ (96–98% in D_2_O, 99.5%) was purchased from Deutero GmbH (Kastellaun, Germany). 4-Fluorobenzoic acid-α-^13^C-2,3,5,6-d4 and 4-trifluoromethyl-benzoic acid-α-^13^C were bought from Sigma-Aldrich (Saint-Quentin-Fallavier, France). Deuterated 2,4-dFBA and 3,4,6-tFBA were synthesized in the lab. A 400 mg of a FBA standard was added to 6 mL of concentrated D_2_SO_4_ in a microwave round-bottom flask and placed in the synthesis microwave oven (CEM, Discover, USA) and heated at 150 °C for 2 or 5 min for 2,4-dFBA and 3,4,6-tFBA, respectively, to obtain doubly deuterated derivatives. To evaluate the efficiency of synthesis the products were diluted in 50:50 ACN/H_2_O and analyzed by direct infusion negative ESI-MS in standard conditions.

### Reagents

The Amberlite MB-20 mixed bed ion-exchange resin was provided by Sigma-Aldrich. Acetonitrile LC-MS grade and acetic acid (purity ≥99%) were acquired from Sigma-Aldrich. Ultrapure water (18 MΩ × cm) was obtained from a Milli-Q system (Millipore, Bedford, MA).

### Sample preparation

Samples were filtered through a 0.2-μm (13-mm) nylon syringe filter, isotopically labeled internal standards 4-fluorobenzoic acid-α-13C-2,3,5,6-d4; 4-trifluoromethyl-benzoic acid-α-13C; 2,4-difluorobenzoic acid-di-2H; and 3,4,5-trifluorobenzoic acid-di-2H were added before filtration with resulting concentration of 20 ng/mL. A sample aliquot (1 mL) was transferred into the test tube containing resin (75 mg) and shaken vigorously for 2 min. A supernatant was recovered after the natural sedimentation. The amount of resin for 1 mL of sample has to be chosen experimentally when the amount of salts is unknown. Otherwise, the amount of ion exchange resin for presented recoveries of FBAs is around 1/3 of total salt content.

### Instrumentation

An Acquity UPLC system (Waters, Milford, MA) including a binary solvent pump, a cooled autosampler, and an Acquity UPLC BEH C18 column, 150 × 2.1 mm (1.7 μm particles, Waters) with a matching Vanguard pre-column was used. The detector was a XevoTQ (quadrupole-T-wave-quadrupole) MS with an orthogonal Z-spray-electrospray interface (Waters).

### HPLC-MS/MS conditions

A 50-μl aliquot was analyzed by HPLC-MS/MS. Mobile phase consisted of 0.05% CH_3_COOH in water (A) and 0.05% CH_3_COOH in acetonitrile (B). The elution gradient was: 0 min (13% B), 1.3 min (13% B), 9 min (28% B), and 13 min (80% B). The column was equilibrated for 5 min. The flow rate was 0.45 mL/min, the column temperature was 45 °C, and the autosampler temperature was 5 °C. MS/MS data acquisition was performed with the electrospray source operating in negative mode (ESI-neg) under the SRM conditions reported elsewhere [7] and listed in Table S1 (see Electronic Supplementary Material, ESM). The electrospray capillary was at 1.4 kV, desolvation temperature was at 550 °C, cone gas flow rate, and desolvation gas flow rate were at 50 and 900 L/h, respectively.

## Results and discussion

### HPLC-MS/MS analysis

**Fig. 1:**
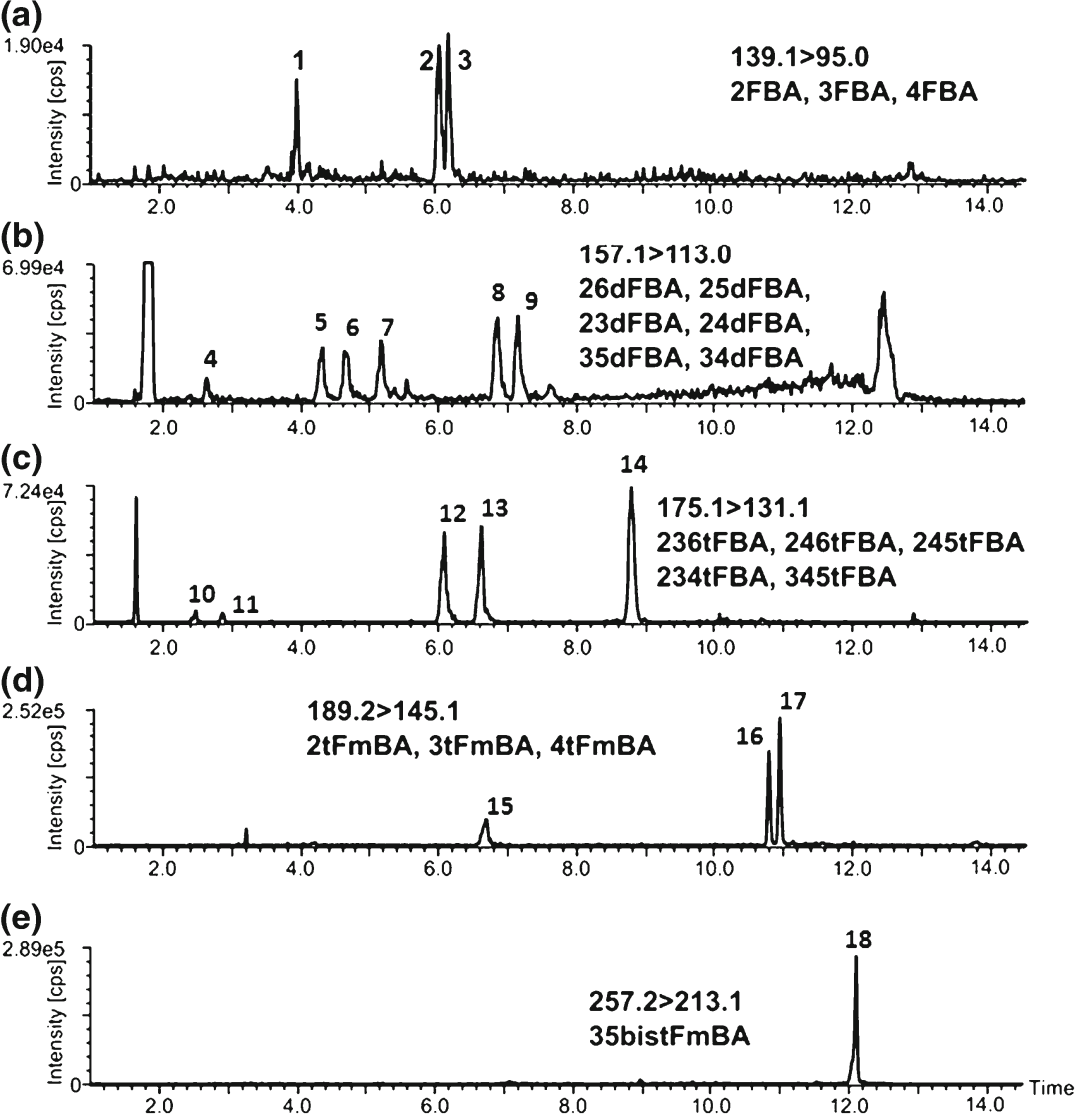
Chromatograms for samples obtained by adding a mixture of standards (at the level of 5 ng/mL) to the reservoir water matrix and analyzed by the developed method. **a** 139 > 95: (*1*) 2-fluorobenzoic acid, (*2*) 3-fluorobenzoic acid, (*3*) 4-fluorobenzoic acid. **b** 157 > 113: (*4*) 2,6-difluorobenzoic acid, (*5*) 2,5-difluorobenzoic acid, (*6*) 2,3-difluorobenzoic acid, (*7*) 2,4-difluorobenzoic acid, (*8*) 3,5-difluorobenzoic acid, (*9*) 3,4-difluorobenzoic acid. **c** 175 > 113: (*10*) 2,3,6-trifluorobenzoic acid, (*11*) 2,4,6-trifluorobenzoic acid, (*12*) 2,4,5-trifluorobenzoic acid, (*13*) 2,3,4-trifluorobenzoic acid, (*14*) 3,4,5-trifluorobenzoic acid. **d** 189 > 145: (*15*) 2-(trifluoromethyl)benzoic acid, (*16*) 3-(trifluoromethyl)benzoic acid, (*17*) 4-(trifluoromethyl)benzoic acid. **e** 257 > 213: (*18*) 3,5-bis(trifluoromethyl)benzoic acid

A typical set of chromatograms for samples obtained by spiking standards on the reservoir water matrix and analyzed by the developed method is illustrated in Fig. 1. In an authentic sample, there are never all the 18 compounds present at the same time; therefore, artificial samples were used to develop a universal method. The absence of the interfering effect of the matrix (both inorganic and organic) was demonstrated by the similar response obtained for the FBA standards spiked on MQ water (results not shown) and for the FBA standards spiked on reservoir water.

### Optimization of the efficiency of the salt removal

Sodium and chlorine ions are responsible for ionization suppression and reduce the sensitivity [3, 12]. Non-volatile sodium or calcium chlorides contaminate the ion source and negatively affect the reproducibility. Theoretically, matrix removal could be accomplished on-line if ion-exchange cartridges with sufficient capacity to handle several samples were available, but they are not. The use of ion-exchange resin was investigated to adsorb the salts while the FBA compounds were supposed to be recovered in the supernatant. The mixed bed type removing anions and cations by the replacement by OH^−^ and H^+^, respectively, was chosen [13–17].

**Fig. 2:**
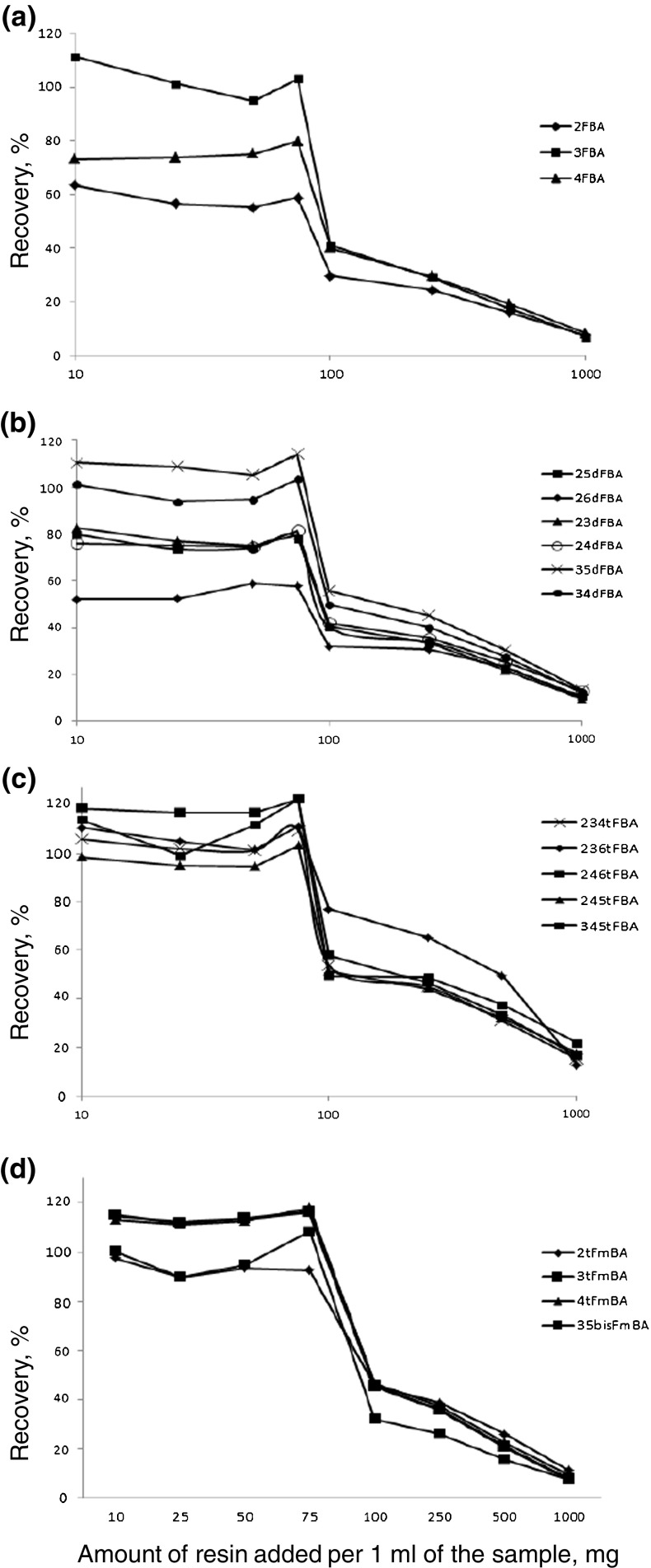
Analytes recoveries obtained during optimization of the matrix removal for **a** mono-FBAs, **b** di-FBAs, **c** tri-FBAs, and **d** triF-methyl-FBAs at 10 ng/mL concentration level for each compound in spiked sample (standard deviation values calculated for 3 measurements were between 3.1 and 5.1%)

The efficiency of the extraction was performed in triplicates using spiked samples with 10 ng/mL of each FBA. The resin addition was controlled with variations not exceeding 15 mg. The method was tested by three independent operators during several months with good results. The most promising results (25–75 mg of resin) were repeated with 1 and 5 ng/mL (data not shown). The recovery was reproducible within 10% for 25–75 mg of resin added. Figure 2 shows the effect of the amount of resin used in the recovery of the 10 ng/mL of FBA tracers (*n* = 3) into the supernatant. It can be seen that up to a certain value (75 mg of resin added), and the recovery of the FBA tracers is stable and superior to 80% for all of the compounds except 2-FBA (ca. 60%) and 2,6d-FBA (ca. 50%). A higher resin/sample ratio results in the rapid decrease of the recovery as the tracers in the ionized form (pH > 3) start competing successfully with the residual salt for the active sites of the resin. An amount of 75 mg of resin for 250 mg of salt was therefore chosen. The pH of acidified samples with formic acid was around 2.40. After addition the 75 mg of resin and shaking the pH dropped to around 2.15–2.30 depending on the sample. The observed drop in the pH could be treated as positive outcome, due to the increased presence of protonated forms of FBAs.

The recoveries were quantitative for the t-FBAs, 2tFm-FBA, tetra-FBA, and 3,5bis-FBA, and d-FBAs not containing F in the *orto* (2) position. Recoveries for the mono- and di-substituted FBAs containing a fluorine atom at 2 positions were at the 80% level. The lowest recovery was for 2,6-dFBA where both fluorine atoms are in the close vicinity of the carboxylic group; this effect needs to be corrected by an internal standard. Also, for 2,4,6-tri-substituted, it appears that the F atoms in the 2 and 6 positions decrease the recovery. It must be further noted that these compounds elute early in the gradient where the conditions of their ionization (and detection) are less favorable than for later-eluting species. The 2,6 substituted compounds were also reported to be the most difficult to derivatize for GC-based determination [8] and quantitatively recovered by SPE [7].

The amount of resin used for sample preparation is around 75 mg. The price of 1 kg of resin is approximately 250 €, which makes the price of one sample preparation (including only resin) to around 2 cents. Price of 1 SPE cartridge depending on volume and amount of sorbent is around 1–2 €. Without online SPE, the presented methods favors the resin over the SPE by time and price. Moreover, the resin method does not require organic solvents for conditioning and cleanup necessary in SPE.

### Quantification: need for isotopically labeled standards

Even if the >80% recoveries can be considered acceptable for the purpose of application, precision and accuracy can be improved by the used of isotopically labeled standards. Hence, 80% recovery of 4-FBA (Fig. 1a) was corrected with 4-fluorobenzoic acid-α-^13^C-2,3,5,6-d^4^; the 80% recoveries of 2,3-dFBA, 2,4-dFBA, and 2,5dFBA were corrected by a 2,4-difluorobenzoic acid-di-^2^H standard; and the 60% recovery of the 2,6dFBA was corrected by 2,4-difluorobenzoic acid-di-^2^H. Even if no correction was judged necessary for the tFBA (Fig. 1c) and tFmBa (Fig. 1d), representative isotopically labeled standards 3,4,5-trifluorobenzoic acid-di-^2^H and 4-trifluoro-methyl-benzoic acid-α-^13^C were used for the purpose of the quality control for each group of compounds. Consequently, all the recoveries could be corrected. An external calibration curve could be used for all the compounds except 2-FBA. For this compound, no isotopically labeled standard was available and matrix matched calibration is required for accurate analysis.

### Figures of merit

The figures of merit of the HPLC-MS/MS analysis are presented in Table 1. The LOD was estimated with the equation LOD = 3.3*S*_*b*_/*a*, where *S*_*b*_ is the standard deviation of the intercept and *a* is the slope of the calibration curve [18]. The criteria to be maintained included LOD < Cmin in calibration curve equation and 10 × LOD > Cmin. The limit of quantitation was calculated as three times the LOD. The recoveries were calculated on the basis of spiked samples at three concentration levels (*n* = 6) using calibration curve equations for each FBA, and statistical data including SD and CV were also calculated. Taking into account the difference in injection volume (50 μL instead of 15 μL) and the dilution factor (no dilution in comparison with 10-fold dilution elsewhere [12], a 37-fold theoretical gain would be expected. In fact, for 4 out of 18 investigated compounds species, the gains are largely superior which means that the method allowed the elimination of the signal suppression factors present during the direct analysis. On the other hand, in some cases, the gain smaller than expected (ca. 10–25×) and virtually non-existent in the case of trifluoromethyl species. This is due to the increase in the background in comparison with the method based on the dilution.

**Table 1:** Linearity, detection, and quantification limits for the method developed applied to a reservoir water (source Quatar, >20% salt) compared with standard direct method [12]

Name	Calibration curve equation for 1/*x* (*n* = 3)	*R* ^2^	LOD [ng/mL]	LOQ [ng/mL]	LOQ (direct method), [ng/mL] [12]	LOQ gain factor^a^	Added [ng/ml]	Found [ng/mL ± SD] (*n* = 6)	CV [%]	Recovery [%]
2-FBA^b^	*y* = 0.11812*x* − 0.011	0.9997	0.09	0.28	4.6	22	0.2	–	–	–
							1.0	0.80 ± 0.05	6	80
							5.0	4.4 ± 0.5	11	88
3-FBA^c^	*y* = 0.21965*x* + 0.2028	0.9999	0.027	0.08	1.1	14	0.2	0.19 ± 0.01	5	96
							1.0	0.93 ± 0.01	1	93
							5.0	4.6 ± 0.5	11	91
4-FBA^c^	*y* = 0.2150*x* + 0.0044	0.9995	0.055	0.16	12	75	0.2	0.16 ± 0.02	13	81
							1.0	0.92 ± 0.03	3	92
							5.0	4.79 ± 0.05	1	96
2,6-dFBA^b^	*y* = 0.1143*x* − 0.0116	0.9970	0.15	0.45	7.3	16	0.2	–	–	–
							1.0	0.91 ± 0.06	7	91
							5.0	5.0 ± 0.1	2	100
2,5-dFBA^c^	*y* = 0.6681*x* + 0.0080	0.9995	0.014	0.041	1.0	24	0.2	0.16 ± 0.02	13	81
							1.0	0.93 ± 0.03	3	93
							5.0	4.8 ± 0.4	8	96
2,3-dFBA^c^	*y* = 0.6824*x* − 0.0111	0.9997	0.020	0.060	0.8	13	0.2	0.18 ± 0.02	11	91
							1.0	0.90 ± 0.04	4	90
							5.0	4.4 ± 0.4	9	88
2,4-dFBA^c^	*y* = 0.6758*x* − 0.0058	0.9995	0.012	0.037	0.9	24	0.2	0.19 ± 0.02	11	94
							1.0	0.97 ± 0.07	7	97
							5.0	4.8 ± 0.1	2	95
3,5-dFBA^c^	*y* = 1.0487*x* + 0.021	0.9997	0.038	0.113	0.2	2	0.2	0.18 ± 0.01	6	92
							1.0	1.03 ± 0.05	5	103
							5.0	4.9 ± 0.4	8	98
3,4-dFBA^c^	*y* = 1.0305*x* + 0.0126	0.9997	0.029	0.086	0.2	2	0.2	0.215 ± 0.01	5	108
							1.0	0.96 ± 0.06	6	96
							5.0	4.6 ± 0.3	7	93
2,3,6-tFBA^b^	*y* = 0.1143*x* − 0.0116	0.9970	0.15	0.45	57	127	0.2	–	–	–
							1.0	0.93 ± 0.07	8	93
							5.0	4.6 ± 0.4	9	92
2,4,6-tFBA^b^	*y* = 0.06804*x* − 0.0120	0.9983	0.14	0.41	21	51	0.2	–	–	–
							1.0	0.91 + 0.08	9	91
							5.0	4.7 ± 0.7	15	94
2,4,5-tFBA^c^	*y* = 1.2872*x* + 0.1541	0.9997	0.010	0.031	4.8	155	0.2	0.164 ± 0.01	6	82
							1.0	0.90 ± 0.04	4	90
							5.0	4.6 ± 0.2	4	92
2,3,4-tFBA^c^	*y* = 1.1548*x* − 0.0190	0.9994	0.011	0.032	4.9	153	0.2	0.17 ± 0.02	12	83
							1.0	0.95 ± 0.07	7	95
							5.0	4.8 ± 0.1	2	96
3,4,5-tFBA^c^	*y* = 1.8497*x* + 0.021	0.9996	0.028	0.084	1.1	13	0.2	0.20 ± 0.02	10	98
							1.0	1.02 ± 0.03	3	102
							5.0	4.7 ± 0.3	6	94
2-tFmBA^d^	*y* = 0.5846*x* − 0.0205	0.9998	0.041	0.123	0.3	2	0.2	0.173 ± 0.003	2	87
							1.0	0.96 ± 0.06	6	96
							5.0	4.6 ± 0.2	4	92
3-tFmBA^c^	*y* = 1.486*x* + 0.074	0.9993	0.073	0.219	0.3	1	0.2	0.22 ± 0.03	14	111
							1.0	1.05 ± 0.01	1	105
							5.0	4.7 ± 0.3	6	95
4-tFmBA^c^	*y* = 1.382*x* + 0.062	0.9993	0.071	0.213	0.2	1	0.2	0.21 ± 0.01	5	106
							1.0	1.01 ± 0.03	3	101
							5.0	4.6 ± 0.3	7	92
3,5-bisFmBA^c^	*y* = 1.612*x* + 0.174	0.9992	0.071	0.213	0.08	–	0.2	0.22 ± 0.02	9	109
							1.0	1.04 ± 0.03	3	104
							5.0	4.7 ± 0.3	6	93

*R* ^*2*^ coefficient of determination, *LOD* limit of detection, *LOQ* limit of quantitation, *n* number of measurements, *CV* coefficient of variation

^a^LOQ comparison with the corresponding value reported for the direct dilution LC MS/MS method [12]

^b^The calibration points were 0.2, 0.5, 1, 10, 20, and 100 ng/ml

^c^The calibration points were 0.05, 0.1, 0.2, 0.5, 1, 10, 20, and 100 ng/ml

^d^The calibration points were 0.1, 0.2, 0.5, 1, 10, 20, and 100 ng/ml

### Validation of the developed method

The validation experiments were carried out following the procedures [19, 20] recommended for similar studies. In order to validate the method, three synthetic samples containing all the tracers at different concentration levels: 0.2, 1, and 5 ng/mL were prepared and analyzed according to the developed procedure with six replicates. All recoveries were calculated against standard solutions of FBAs. The results presented in Table 1 demonstrate high and consistent recoveries. The only exceptions were early eluting compounds—2,6-dFBA; 2,3,6-tFBA; and 2,4,6-tFBA—due to the lower detection limits and 2-FBA that has not been corrected because of the lack of the internal standard.

**Fig. 3:**
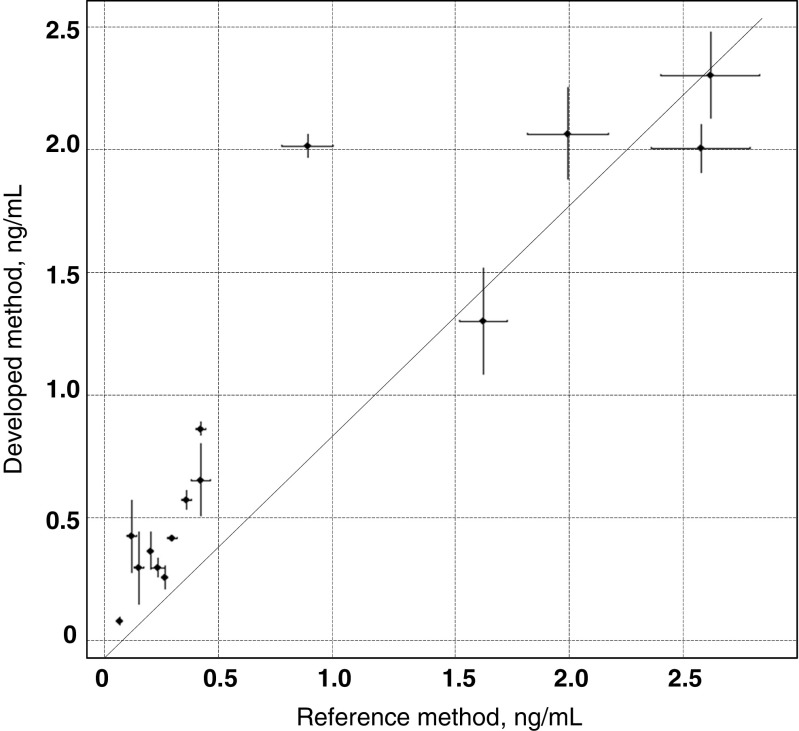
Validation with SPE-LC MS/MS; the points on the graph (with *error bars*) correspond to concentration values obtained by the proposed method and according to the literature method [7] for 10 reservoir water samples

An additional validation was achieved for 10 authentic reservoir water samples by an independent comparison (different day, different operator) with the method based on the SPE-HPLC MS/MS reported elsewhere [7]. The results shown in Fig. 3 present good correlation of the data; the Pearson product-moment correlation coefficient (which is a measure of the linear correlation between the two variables) is 0.907101 with a *p* value of 3.072 × 10^−6^. The linear relationship between two data sets is described by the following: *y* = 1.02520*x* − 0.09935.

## Electronic supplementary material

Below is the link to the electronic supplementary material.

ESM 1 (PDF 94 kb)
