# Supplementary material for: Rapid ion-exchange matrix removal for a decrease of detection limits in the analysis of salt-rich reservoir waters for fluorobenzoic acids by liquid chromatography coupled with tandem mass spectrometry
Source: Anal Bioanal Chem. 2016 Nov 10;409(4):871–9. doi: 10.1007/s00216-016-0060-5 (PMC5258790; doi:10.1007/s00216-016-0060-5)
Supplement: Supplementary file 1 — (PDF 94 kb) [file 216_2016_60_MOESM1_ESM.pdf]

Analytical and Bioanalytical Chemistry

Electronic Supplementary Material

Rapid ion-exchange matrix removal for a decrease of detection limits in the analysis of salt-rich reservoir waters for fluorobenzoic acids by liquid chromatography coupled with tandem mass spectrometry

Paweł Kubica, Véronique Vacchina, Tomasz Wasilewski, Stéphanie Reynaud, Joanna Szpunar, Ryszard Lobinski

Table S1 MRM transitions parameters and operating parameters of ESI ion source

**Name**	**Formula**	**Mass**	**Ion transition**	**Cone [V]**	**Collision [V]**
2-FBA	$C_7H_5O_2F$	140.11	139.1 -> 95.0	22	12
3-FBA	$C_7H_5O_2F$	140.11		18	10
4-FBA	$C_7H_5O_2F$	140.11		22	12
4-FBA-α-^{13}C-2,3,5,6-d^4	$C_6D_4{}^{13}CO_2HF$	145.13	144.0 -> 99.1	20	14
2,3-dFBA	$C_7H_4O_2F_2$	158.10	157.1 -> 113.0	14	12
2,4-dFBA	$C_7H_4O_2F_2$	158.10		16	10
2,6-dFBA	$C_7H_4O_2F_2$	158.10		10	8
2,5-dFBA	$C_7H_4O_2F_2$	158.10		14	10
3,4-dFBA	$C_7H_4O_2F_2$	158.10		20	14
3,5-dFBA	$C_7H_4O_2F_2$	158.10		14	10
2,4-dFBA-d^2	$C_7H_2D_2O_2F_2$	160.10	159.1 -> 115.0	14	10
2,3,4-tFBA	$C_7H_3O_2F_3$	176.10	175.1 -> 131.1	14	12
2,3,6-tFBA	$C_7H_3O_2F_3$	176.10		12	8
2,4,5-tFBA	$C_7H_3O_2F_3$	176.10		14	12
2,4,6-tFBA	$C_7H_3O_2F_3$	176.10		10	8
3,4,5-tFBA	$C_7H_3O_2F_3$	176.10		20	12
3,4,6-tFBA-d^2	$C_7H_3O_2F_3$	178.10	177.1 -> 133.1	20	12
2-tFmBA	$C_9H_5O_2F_3$	190.12	189.2 -> 145.1	20	12
3-tFmBA	$C_9H_5O_2F_3$	190.12		26	14
4-tFmBA	$C_9H_5O_2F_3$	190.12		22	14
3,5-bistFmBA	$C_9H_4O_2F_6$	258.12	257.2 -> 213.1	22	16
4-tFmBA-αC^{13}	$C_8H_5{}^{13}CO_2F_3$	191.12	190.1 -> 145.1	22	14

Ion source parameters

Capillary [kV]	Desolvation temp. [˚C]	Cone gas [L/h]	Desolvation gas [L/h]
1.4	550	50	900
